# *N*′-(3-Methoxy­benzyl­idene)-4-nitro­benzohydrazide monohydrate

**DOI:** 10.1107/S1600536810010986

**Published:** 2010-03-31

**Authors:** Tanveer Ahmad, Muhammad Zia-ur-Rehman, Hamid Latif Siddiqui, Muhammad Fasih Ullah, Masood Parvez

**Affiliations:** a Institute of Chemistry, University of the Punjab, Lahore 54590, Pakistan; b Applied Chemistry Research Centre, PCSIR Laboratories Complex, Lahore 54600, Pakistan; c Department of Chemistry, The University of Calgary, 2500 University Drive NW, Calgary, Alberta, Canada T2N 1N4

## Abstract

There are two independent formula units in the asymmetric unit of the title compound, C_15_H_13_N_3_O_4_·H_2_O. The C=C—N—C torsion angle of the methyl­idenehydrazide group is 174.3 (2)° in one mol­ecule and 178.6 (2)° in the other. The dihedral angles between the two benzene rings in the two mol­ecules are 4.17 (12) and 3.58 (12)°. In the crystal structure, inter­molecular O—H⋯O, N—H⋯O and O—H⋯N hydrogen bonds link the components into a two-dimensional network and additional stabilization is provided by weak inter­molecular C—H⋯O hydrogen bonds.

## Related literature

For the synthesis of related compounds, see: Zia-ur-Rehman *et al.* (2005 ▶, 2006 ▶). For the biological activity of benzohydrazides, see: Zia-ur-Rehman *et al.* (2009 ▶); Jiang *et al.* (1990 ▶); Ochiai & Ishida (1982 ▶); Guersoy *et al.* (1995 ▶); Farghaly & Moharram (1999 ▶). For related structures, see: Raj *et al.* (2008 ▶); Fun *et al.* (2008 ▶); Wang *et al.* (2008 ▶); Qiu *et al.* (2009 ▶).
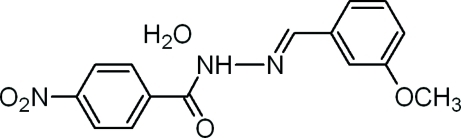


## Experimental

### 

#### Crystal data

C_15_H_13_N_3_O_4_·H_2_O
*M* *_r_* = 317.30
Triclinic, 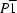

*a* = 6.7162 (3) Å
*b* = 7.4929 (3) Å
*c* = 32.1141 (15) Å
α = 91.883 (2)°
β = 91.5697 (12)°
γ = 112.753 (2)°
*V* = 1488.12 (11) Å^3^
*Z* = 4
Mo *K*α radiation
μ = 0.11 mm^−1^
*T* = 173 K
0.10 × 0.04 × 0.03 mm

#### Data collection

Nonius KappaCCD diffractometer
Absorption correction: multi-scan (*SORTAV*; Blessing, 1997 ▶) *T*_min_ = 0.989, *T*_max_ = 0.997
10724 measured reflections
6546 independent reflections
5035 reflections with *I* > 2σ(*I*)
*R*_int_ = 0.035

#### Refinement

*R*[*F* ^2^ > 2σ(*F* ^2^)] = 0.071
*wR*(*F* ^2^) = 0.149
*S* = 1.17
6546 reflections
429 parameters
H atoms treated by a mixture of independent and constrained refinement
Δρ_max_ = 0.30 e Å^−3^
Δρ_min_ = −0.24 e Å^−3^

### 

Data collection: *COLLECT* (Hooft, 1998 ▶); cell refinement: *DENZO* (Otwinowski & Minor, 1997 ▶); data reduction: *SCALEPACK* (Otwinowski & Minor, 1997 ▶); program(s) used to solve structure: *SHELXS97* (Sheldrick, 2008 ▶); program(s) used to refine structure: *SHELXL97* (Sheldrick, 2008 ▶); molecular graphics: *ORTEP-3 for Windows* (Farrugia, 1997 ▶); software used to prepare material for publication: *SHELXL97*.

## Supplementary Material

Crystal structure: contains datablocks global, I. DOI: 10.1107/S1600536810010986/lh5018sup1.cif

Structure factors: contains datablocks I. DOI: 10.1107/S1600536810010986/lh5018Isup2.hkl

Additional supplementary materials:  crystallographic information; 3D view; checkCIF report

## Figures and Tables

**Table 1:** Hydrogen-bond geometry (Å, °)

*D*—H⋯*A*	*D*—H	H⋯*A*	*D*⋯*A*	*D*—H⋯*A*
N2—H2*N*⋯O9	0.88	1.98	2.839 (3)	166
N5—H5*N*⋯O10	0.88	2.08	2.930 (3)	163
C2—H2⋯O9	0.95	2.23	3.153 (3)	164
C8—H8⋯O9	0.95	2.45	3.277 (3)	145
C15—H15*B*⋯O6	0.98	2.55	3.443 (4)	151
C21—H21⋯O10	0.95	2.42	3.355 (3)	169
C23—H23⋯O10	0.95	2.46	3.295 (3)	147
O9—H9*A*⋯O7^i^	0.91 (4)	2.26 (3)	3.020 (3)	140 (3)
O9—H9*A*⋯N6^i^	0.91 (4)	2.34 (3)	3.093 (3)	140 (3)
O9—H9*B*⋯O3^i^	0.92 (3)	1.84 (4)	2.758 (3)	173 (3)
O10—H10*A*⋯O7^i^	0.87 (4)	1.99 (4)	2.846 (3)	167 (3)
O10—H10*B*⋯O3^ii^	0.80 (4)	2.17 (4)	2.925 (3)	158 (3)
C13—H13⋯O4^i^	0.95	2.52	3.390 (3)	153
C15—H15*A*⋯O6^iii^	0.98	2.59	3.560 (4)	171
C30—H30*A*⋯O2^iv^	0.98	2.40	3.322 (4)	156
C30—H30*B*⋯O1^v^	0.98	2.59	3.200 (4)	121

Symmetry codes: (i) 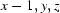; (ii) 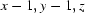; (iii) 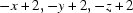; (iv) 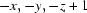; (v) 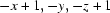.

**Fig. 1.**
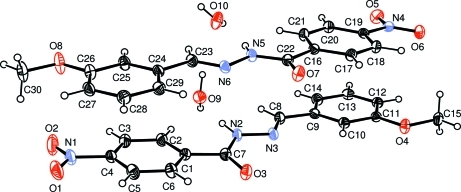
The asymmetric unit of the title compound with the displacement ellipsoids plotted at 30% probability level (Farrugia, 1997).

## supplementary crystallographic information

### Comment

Benzohydrazides and their derivatives are reported to show a wide variety of biological activities. For example, some of these are found useful for the treatment of autoimmune and inflammatory diseases, tumors, osteoarthritis and hemorrhage (Jiang *et al.*, 1990) whereas some others exhibit antibacterial and anti-oxidant (Zia-ur-Rehman *et al.*, 2009), anti-viral (Ochiai & Ishida, 1982), anti-tuberculous (Guersoy *et al.*, 1995) and insecticidal activities (Farghaly & Moharram, 1999). As part of our on-going research on the synthesis of various heterocyclic compounds (Zia-ur-Rehman *et al.*, 2005; 2006; 2009), we herein report the crystal structure of the title compound.

The structure of the title compound is composed of two independent molecules and two molecules of water of hydration (Fig. 1) in an asymmetric unit. The bond distances and angles agree with the cortresponding bond distances and angles reported in closely related compounds (Raj *et al.*, 2008; Fun *et al.*, 2008; Wang *et al.*, 2008; Qiu *et al*., 2009). The methylidenehydrazide fragment C22/C23/N5/N6/O7 is essentially planar with the maximum deviation of N6 being 0.0194 (18) Å compared to the corresponding fragment in the other molecule (C7/C8/N2/N3/O3) wherein C8 and N3 deviate by 0.0411 (14) and 0.0501 (18) Å in opposite directions from the mean-planes formed by these atoms. The mean-planes of the phenyl rings C1–C6 and C9–C14 make dihedral angles of 8.83 (14) and 12.24 (12)°, respectively, with the mean-plane of the methylidenehydrazide fragment; the corresponding dihedral angles in the other molecule are 3.23 (14) and 0.90 (14)°, respectively. The C═C—N—C torsion angle of the methylidenehydrazide group is 174.3 (2)° in one molecule and 178.6 (2)° in the other. The dihedral angle between the two benzene rings in each molecule are 4.17 (12) and 3.58 (12)°. In the crystal structure, intermolecular O—H..O, N—H..O and O—H···N hydrogen bonds link the components of the structure into a two-dimensional network and additional stabilization is provided by weak intermolecular C—H···O hydrogen bonds; geometric details are provided in Table 1.

### Experimental

A mixture of *p*-nitrobenzohydrazide (0.1 g; 0.552 moles), 4-methoxybenzaldehyde (0.33 ml; 0.552 mmoles), orthophosphoric acid (0.2 ml) and methanol (50.0 ml) was heated to reflux for 4 hours followed by removal of the solvent under vacuum. The contents were allowed to cool and washed with a mixture of cold methanol-water (9:1) to yield the title compound. Crystals suitable for X-ray crystallographic studies were grown from a mixture of methanol-water (9:1) at room temperature by slow evaporation. Yield: 89%. M.p. 506 K.

### Refinement

Though all the H atoms could be distinguished in the difference Fourier map the H-atoms bonded to C-atoms were included at geometrically idealized positions and refined in riding-model approximation with N—H = 0.88 Å and C—H = 0.95 and 0.98 Å, for aryl and methyl H-atoms, respectively; the H-atoms of the water of hydrate molecules were allowed to refine. The *U*_iso_(H) were allowed at 1.2*U*_eq_(parent atom). The final difference map was essentially featurless.

### Crystal data

**Table:**

C_15_H_13_N_3_O_4_·H_2_O	*Z* = 4
*M**_r_* = 317.30	*F*(000) = 664
Triclinic, *P*1	*D*_x_ = 1.416 Mg m^−^^3^
Hall symbol: -P 1	Melting point: 506 K
*a* = 6.7162 (3) Å	Mo *K*α radiation, λ = 0.71073 Å
*b* = 7.4929 (3) Å	Cell parameters from 5661 reflections
*c* = 32.1141 (15) Å	θ = 1.0–27.5°
α = 91.883 (2)°	µ = 0.11 mm^−^^1^
β = 91.5697 (12)°	*T* = 173 K
γ = 112.753 (2)°	Needle, yellow
*V* = 1488.12 (11) Å^3^	0.10 × 0.04 × 0.03 mm

### Data collection

**Table:**

Nonius KappaCCD diffractometer	6546 independent reflections
Radiation source: fine-focus sealed tube	5035 reflections with *I* > 2σ(*I*)
graphite	*R*_int_ = 0.035
ω and φ scans	θ_max_ = 27.5°, θ_min_ = 3.0°
Absorption correction: multi-scan (*SORTAV*; Blessing, 1997)	*h* = −8→8
*T*_min_ = 0.989, *T*_max_ = 0.997	*k* = −9→9
10724 measured reflections	*l* = −41→41

### Refinement

**Table:**

Refinement on *F*^2^	Primary atom site location: structure-invariant direct methods
Least-squares matrix: full	Secondary atom site location: difference Fourier map
*R*[*F*^2^ > 2σ(*F*^2^)] = 0.071	Hydrogen site location: difference Fourier map
*wR*(*F*^2^) = 0.149	H atoms treated by a mixture of independent and constrained refinement
*S* = 1.17	*w* = 1/[σ^2^(*F*_o_^2^) + (0.0216*P*)^2^ + 1.6183*P*] where *P* = (*F*_o_^2^ + 2*F*_c_^2^)/3
6546 reflections	(Δ/σ)_max_ < 0.001
429 parameters	Δρ_max_ = 0.30 e Å^−^^3^
0 restraints	Δρ_min_ = −0.24 e Å^−^^3^

### Special details

**Table:**

Geometry. All esds (except the esd in the dihedral angle between two l.s. planes) are estimated using the full covariance matrix. The cell esds are taken into account individually in the estimation of esds in distances, angles and torsion angles; correlations between esds in cell parameters are only used when they are defined by crystal symmetry. An approximate (isotropic) treatment of cell esds is used for estimating esds involving l.s. planes.
Refinement. Refinement of *F*^2^ against ALL reflections. The weighted *R*-factor wR and goodness of fit *S* are based on *F*^2^, conventional *R*-factors *R* are based on *F*, with *F* set to zero for negative *F*^2^. The threshold expression of *F*^2^ > σ(*F*^2^) is used only for calculating *R*-factors(gt) etc. and is not relevant to the choice of reflections for refinement. *R*-factors based on *F*^2^ are statistically about twice as large as those based on *F*, and *R*- factors based on ALL data will be even larger.

### Fractional atomic coordinates and isotropic or equivalent isotropic displacement parameters (Å^2^)

**Table:**

	*x*	*y*	*z*	*U*_iso_*/*U*_eq_	
O1	0.5409 (5)	0.3361 (4)	0.52359 (7)	0.0652 (7)	
O2	0.2135 (5)	0.3034 (4)	0.53490 (8)	0.0782 (9)	
O3	0.8790 (3)	0.8188 (3)	0.71569 (6)	0.0423 (5)	
O4	0.8847 (3)	1.1025 (3)	0.92010 (6)	0.0383 (5)	
O5	0.2433 (3)	0.6052 (3)	0.96336 (6)	0.0461 (5)	
O6	0.5729 (3)	0.7096 (3)	0.98840 (6)	0.0432 (5)	
O7	0.8929 (3)	0.4098 (3)	0.80674 (6)	0.0390 (5)	
O8	0.1740 (4)	−0.1127 (4)	0.57904 (6)	0.0613 (7)	
O9	0.0742 (3)	0.5580 (3)	0.72390 (7)	0.0437 (5)	
H9A	−0.016 (5)	0.464 (5)	0.7397 (10)	0.052*	
H9B	0.017 (5)	0.651 (5)	0.7228 (10)	0.052*	
O10	0.0793 (3)	0.1569 (3)	0.77316 (7)	0.0468 (6)	
H10A	0.002 (6)	0.218 (5)	0.7828 (11)	0.056*	
H10B	0.005 (6)	0.052 (5)	0.7631 (11)	0.056*	
N1	0.4053 (5)	0.3579 (4)	0.54538 (8)	0.0522 (7)	
N2	0.5317 (4)	0.7257 (3)	0.73434 (6)	0.0327 (5)	
H2N	0.3936	0.6645	0.7272	0.039*	
N3	0.5961 (4)	0.8154 (3)	0.77402 (6)	0.0327 (5)	
N4	0.4328 (4)	0.6279 (3)	0.96079 (7)	0.0334 (5)	
N5	0.5518 (3)	0.2963 (3)	0.77803 (6)	0.0293 (5)	
H5N	0.4142	0.2727	0.7811	0.035*	
N6	0.6228 (3)	0.2498 (3)	0.74060 (6)	0.0305 (5)	
C1	0.6020 (4)	0.6333 (4)	0.66505 (8)	0.0317 (6)	
C2	0.3846 (5)	0.5612 (4)	0.65178 (8)	0.0379 (6)	
H2	0.2801	0.5730	0.6698	0.045*	
C3	0.3205 (5)	0.4724 (4)	0.61237 (9)	0.0424 (7)	
H3	0.1729	0.4241	0.6030	0.051*	
C4	0.4752 (5)	0.4557 (4)	0.58715 (8)	0.0379 (6)	
C5	0.6907 (5)	0.5251 (4)	0.59902 (9)	0.0420 (7)	
H5	0.7938	0.5119	0.5808	0.050*	
C6	0.7534 (5)	0.6152 (4)	0.63848 (8)	0.0401 (7)	
H6	0.9017	0.6650	0.6474	0.048*	
C7	0.6827 (4)	0.7339 (4)	0.70724 (8)	0.0329 (6)	
C8	0.4388 (4)	0.8124 (4)	0.79569 (8)	0.0343 (6)	
H8	0.2963	0.7536	0.7836	0.041*	
C9	0.4687 (4)	0.8960 (4)	0.83847 (8)	0.0297 (5)	
C10	0.6675 (4)	0.9670 (4)	0.86004 (8)	0.0291 (5)	
H10	0.7925	0.9669	0.8469	0.035*	
C11	0.6828 (4)	1.0387 (3)	0.90111 (8)	0.0272 (5)	
C12	0.5013 (4)	1.0433 (3)	0.92029 (8)	0.0298 (5)	
H12	0.5129	1.0940	0.9482	0.036*	
C13	0.3032 (4)	0.9734 (4)	0.89832 (8)	0.0333 (6)	
H13	0.1787	0.9760	0.9112	0.040*	
C14	0.2862 (4)	0.8998 (4)	0.85770 (8)	0.0350 (6)	
H14	0.1501	0.8517	0.8428	0.042*	
C15	0.9058 (5)	1.1692 (4)	0.96298 (8)	0.0408 (7)	
H15A	1.0560	1.2070	0.9732	0.049*	
H15B	0.8110	1.0650	0.9796	0.049*	
H15C	0.8649	1.2811	0.9655	0.049*	
C16	0.6180 (4)	0.4345 (3)	0.84904 (7)	0.0259 (5)	
C17	0.7721 (4)	0.5258 (4)	0.88126 (8)	0.0299 (5)	
H17	0.9191	0.5459	0.8777	0.036*	
C18	0.7132 (4)	0.5871 (4)	0.91830 (8)	0.0312 (6)	
H18	0.8175	0.6487	0.9403	0.037*	
C19	0.4983 (4)	0.5564 (3)	0.92247 (7)	0.0272 (5)	
C20	0.3404 (4)	0.4629 (4)	0.89158 (8)	0.0302 (5)	
H20	0.1932	0.4408	0.8956	0.036*	
C21	0.4029 (4)	0.4028 (4)	0.85474 (8)	0.0309 (5)	
H21	0.2973	0.3390	0.8331	0.037*	
C22	0.6997 (4)	0.3791 (3)	0.80989 (8)	0.0289 (5)	
C23	0.4724 (4)	0.1688 (4)	0.71271 (8)	0.0323 (6)	
H23	0.3278	0.1467	0.7192	0.039*	
C24	0.5146 (4)	0.1086 (4)	0.67096 (8)	0.0310 (5)	
C25	0.3371 (4)	0.0256 (4)	0.64343 (8)	0.0366 (6)	
H25	0.1975	0.0102	0.6521	0.044*	
C26	0.3616 (5)	−0.0352 (4)	0.60320 (8)	0.0398 (7)	
C27	0.5630 (5)	−0.0151 (4)	0.59032 (8)	0.0374 (6)	
H27	0.5803	−0.0569	0.5629	0.045*	
C28	0.7408 (5)	0.0676 (4)	0.61821 (9)	0.0388 (6)	
H28	0.8799	0.0810	0.6096	0.047*	
C29	0.7193 (4)	0.1306 (4)	0.65817 (8)	0.0361 (6)	
H29	0.8423	0.1881	0.6767	0.043*	
C30	0.1873 (6)	−0.1784 (6)	0.53710 (9)	0.0624 (10)	
H30A	0.0433	−0.2282	0.5232	0.075*	
H30B	0.2426	−0.2817	0.5376	0.075*	
H30C	0.2851	−0.0702	0.5219	0.075*	

### Atomic displacement parameters (Å^2^)

**Table:**

	*U*^11^	*U*^22^	*U*^33^	*U*^12^	*U*^13^	*U*^23^
O1	0.101 (2)	0.0737 (16)	0.0305 (12)	0.0446 (15)	0.0065 (12)	−0.0088 (11)
O2	0.0780 (19)	0.097 (2)	0.0421 (14)	0.0186 (16)	−0.0182 (13)	−0.0240 (14)
O3	0.0343 (10)	0.0517 (12)	0.0369 (11)	0.0137 (9)	−0.0009 (8)	−0.0129 (9)
O4	0.0299 (10)	0.0522 (12)	0.0313 (10)	0.0153 (9)	−0.0018 (8)	−0.0114 (8)
O5	0.0418 (12)	0.0508 (12)	0.0417 (12)	0.0136 (10)	0.0152 (9)	−0.0073 (9)
O6	0.0554 (13)	0.0420 (11)	0.0260 (10)	0.0132 (10)	−0.0029 (9)	−0.0087 (8)
O7	0.0277 (10)	0.0500 (12)	0.0368 (11)	0.0131 (9)	0.0046 (8)	−0.0116 (9)
O8	0.0517 (13)	0.106 (2)	0.0288 (11)	0.0356 (13)	−0.0095 (9)	−0.0207 (12)
O9	0.0313 (11)	0.0545 (13)	0.0409 (12)	0.0114 (10)	0.0061 (9)	0.0046 (10)
O10	0.0312 (11)	0.0570 (14)	0.0513 (13)	0.0186 (10)	−0.0053 (9)	−0.0228 (11)
N1	0.080 (2)	0.0484 (15)	0.0269 (13)	0.0243 (15)	−0.0034 (14)	−0.0049 (11)
N2	0.0337 (12)	0.0380 (12)	0.0236 (11)	0.0117 (10)	−0.0011 (9)	−0.0041 (9)
N3	0.0393 (12)	0.0369 (12)	0.0222 (11)	0.0156 (10)	0.0006 (9)	−0.0025 (9)
N4	0.0437 (13)	0.0256 (11)	0.0280 (11)	0.0102 (10)	0.0080 (10)	0.0002 (9)
N5	0.0288 (11)	0.0379 (12)	0.0230 (10)	0.0150 (9)	0.0043 (8)	−0.0028 (9)
N6	0.0374 (12)	0.0336 (11)	0.0226 (10)	0.0162 (10)	0.0052 (9)	−0.0008 (9)
C1	0.0368 (14)	0.0290 (13)	0.0279 (13)	0.0114 (11)	−0.0001 (11)	−0.0014 (10)
C2	0.0387 (15)	0.0423 (15)	0.0287 (14)	0.0116 (12)	0.0041 (11)	−0.0036 (11)
C3	0.0409 (16)	0.0458 (16)	0.0327 (15)	0.0091 (13)	−0.0029 (12)	−0.0033 (12)
C4	0.0546 (18)	0.0320 (14)	0.0252 (13)	0.0150 (13)	0.0005 (12)	−0.0008 (11)
C5	0.0532 (18)	0.0494 (17)	0.0306 (15)	0.0281 (15)	0.0067 (13)	−0.0038 (12)
C6	0.0425 (16)	0.0495 (17)	0.0310 (14)	0.0211 (14)	0.0007 (12)	−0.0029 (12)
C7	0.0354 (14)	0.0332 (14)	0.0304 (13)	0.0140 (11)	−0.0003 (11)	−0.0018 (11)
C8	0.0347 (14)	0.0392 (15)	0.0276 (13)	0.0132 (12)	−0.0019 (11)	−0.0017 (11)
C9	0.0355 (14)	0.0272 (12)	0.0263 (12)	0.0119 (10)	0.0039 (10)	0.0028 (10)
C10	0.0310 (13)	0.0314 (13)	0.0261 (13)	0.0132 (10)	0.0055 (10)	0.0006 (10)
C11	0.0282 (12)	0.0256 (12)	0.0276 (12)	0.0103 (10)	0.0017 (10)	0.0016 (10)
C12	0.0362 (14)	0.0286 (12)	0.0260 (12)	0.0140 (11)	0.0055 (10)	−0.0008 (10)
C13	0.0301 (13)	0.0369 (14)	0.0353 (14)	0.0148 (11)	0.0100 (11)	0.0041 (11)
C14	0.0330 (14)	0.0400 (15)	0.0329 (14)	0.0152 (12)	0.0006 (11)	0.0021 (11)
C15	0.0406 (16)	0.0427 (16)	0.0296 (14)	0.0068 (13)	−0.0042 (12)	−0.0069 (12)
C16	0.0281 (12)	0.0242 (12)	0.0255 (12)	0.0102 (10)	0.0019 (10)	0.0009 (9)
C17	0.0251 (12)	0.0325 (13)	0.0311 (13)	0.0102 (10)	0.0005 (10)	−0.0014 (10)
C18	0.0299 (13)	0.0330 (13)	0.0276 (13)	0.0096 (11)	−0.0049 (10)	−0.0044 (10)
C19	0.0349 (13)	0.0249 (12)	0.0216 (11)	0.0112 (10)	0.0050 (10)	0.0009 (9)
C20	0.0266 (12)	0.0352 (13)	0.0287 (13)	0.0121 (11)	0.0022 (10)	−0.0007 (10)
C21	0.0270 (12)	0.0380 (14)	0.0250 (12)	0.0103 (11)	−0.0007 (10)	−0.0045 (10)
C22	0.0312 (13)	0.0280 (12)	0.0275 (13)	0.0116 (10)	0.0025 (10)	−0.0009 (10)
C23	0.0362 (14)	0.0358 (14)	0.0265 (13)	0.0158 (11)	0.0044 (11)	−0.0011 (11)
C24	0.0405 (14)	0.0313 (13)	0.0229 (12)	0.0154 (11)	0.0038 (10)	0.0014 (10)
C25	0.0357 (14)	0.0471 (16)	0.0288 (14)	0.0183 (12)	0.0037 (11)	−0.0018 (12)
C26	0.0460 (16)	0.0493 (17)	0.0261 (14)	0.0214 (14)	−0.0036 (12)	−0.0041 (12)
C27	0.0512 (17)	0.0396 (15)	0.0241 (13)	0.0204 (13)	0.0061 (12)	0.0000 (11)
C28	0.0413 (16)	0.0403 (15)	0.0345 (15)	0.0151 (13)	0.0104 (12)	−0.0006 (12)
C29	0.0371 (15)	0.0376 (14)	0.0323 (14)	0.0131 (12)	0.0051 (11)	−0.0010 (11)
C30	0.065 (2)	0.092 (3)	0.0277 (16)	0.030 (2)	−0.0091 (15)	−0.0170 (17)

### Geometric parameters (Å, °)

**Table:**

O1—N1	1.220 (4)	C9—C14	1.397 (4)
O2—N1	1.225 (4)	C10—C11	1.393 (3)
O3—C7	1.240 (3)	C10—H10	0.9500
O4—C11	1.368 (3)	C11—C12	1.393 (3)
O4—C15	1.433 (3)	C12—C13	1.388 (4)
O5—N4	1.223 (3)	C12—H12	0.9500
O6—N4	1.233 (3)	C13—C14	1.384 (4)
O7—C22	1.235 (3)	C13—H13	0.9500
O8—C26	1.370 (3)	C14—H14	0.9500
O8—C30	1.436 (3)	C15—H15A	0.9800
O9—H9A	0.91 (4)	C15—H15B	0.9800
O9—H9B	0.92 (3)	C15—H15C	0.9800
O10—H10A	0.87 (4)	C16—C21	1.389 (3)
O10—H10B	0.80 (4)	C16—C17	1.399 (3)
N1—C4	1.480 (3)	C16—C22	1.495 (3)
N2—C7	1.339 (3)	C17—C18	1.383 (3)
N2—N3	1.397 (3)	C17—H17	0.9500
N2—H2N	0.8800	C18—C19	1.382 (4)
N3—C8	1.274 (3)	C18—H18	0.9500
N4—C19	1.471 (3)	C19—C20	1.386 (3)
N5—C22	1.358 (3)	C20—C21	1.385 (3)
N5—N6	1.388 (3)	C20—H20	0.9500
N5—H5N	0.8800	C21—H21	0.9500
N6—C23	1.277 (3)	C23—C24	1.470 (3)
C1—C6	1.388 (4)	C23—H23	0.9500
C1—C2	1.395 (4)	C24—C25	1.386 (4)
C1—C7	1.508 (3)	C24—C29	1.395 (4)
C2—C3	1.387 (4)	C25—C26	1.391 (4)
C2—H2	0.9500	C25—H25	0.9500
C3—C4	1.375 (4)	C26—C27	1.379 (4)
C3—H3	0.9500	C27—C28	1.395 (4)
C4—C5	1.373 (4)	C27—H27	0.9500
C5—C6	1.391 (4)	C28—C29	1.384 (4)
C5—H5	0.9500	C28—H28	0.9500
C6—H6	0.9500	C29—H29	0.9500
C8—C9	1.467 (3)	C30—H30A	0.9800
C8—H8	0.9500	C30—H30B	0.9800
C9—C10	1.386 (4)	C30—H30C	0.9800
			
C11—O4—C15	117.2 (2)	C13—C14—C9	120.1 (2)
C26—O8—C30	117.7 (2)	C13—C14—H14	119.9
H9A—O9—H9B	106 (3)	C9—C14—H14	119.9
H10A—O10—H10B	112 (3)	O4—C15—H15A	109.5
O1—N1—O2	124.0 (3)	O4—C15—H15B	109.5
O1—N1—C4	118.4 (3)	H15A—C15—H15B	109.5
O2—N1—C4	117.5 (3)	O4—C15—H15C	109.5
C7—N2—N3	119.2 (2)	H15A—C15—H15C	109.5
C7—N2—H2N	120.4	H15B—C15—H15C	109.5
N3—N2—H2N	120.4	C21—C16—C17	119.3 (2)
C8—N3—N2	113.4 (2)	C21—C16—C22	124.1 (2)
O5—N4—O6	123.5 (2)	C17—C16—C22	116.5 (2)
O5—N4—C19	118.4 (2)	C18—C17—C16	120.8 (2)
O6—N4—C19	118.1 (2)	C18—C17—H17	119.6
C22—N5—N6	118.4 (2)	C16—C17—H17	119.6
C22—N5—H5N	120.8	C19—C18—C17	118.1 (2)
N6—N5—H5N	120.8	C19—C18—H18	120.9
C23—N6—N5	114.2 (2)	C17—C18—H18	120.9
C6—C1—C2	119.4 (2)	C18—C19—C20	122.7 (2)
C6—C1—C7	117.7 (2)	C18—C19—N4	119.2 (2)
C2—C1—C7	122.9 (2)	C20—C19—N4	118.1 (2)
C3—C2—C1	120.3 (3)	C21—C20—C19	118.3 (2)
C3—C2—H2	119.9	C21—C20—H20	120.9
C1—C2—H2	119.9	C19—C20—H20	120.9
C4—C3—C2	118.5 (3)	C20—C21—C16	120.8 (2)
C4—C3—H3	120.7	C20—C21—H21	119.6
C2—C3—H3	120.7	C16—C21—H21	119.6
C5—C4—C3	123.0 (3)	O7—C22—N5	122.0 (2)
C5—C4—N1	118.7 (3)	O7—C22—C16	121.2 (2)
C3—C4—N1	118.3 (3)	N5—C22—C16	116.7 (2)
C4—C5—C6	118.0 (3)	N6—C23—C24	122.5 (2)
C4—C5—H5	121.0	N6—C23—H23	118.8
C6—C5—H5	121.0	C24—C23—H23	118.8
C1—C6—C5	120.8 (3)	C25—C24—C29	119.6 (2)
C1—C6—H6	119.6	C25—C24—C23	116.5 (2)
C5—C6—H6	119.6	C29—C24—C23	123.9 (2)
O3—C7—N2	122.9 (2)	C24—C25—C26	120.6 (3)
O3—C7—C1	120.7 (2)	C24—C25—H25	119.7
N2—C7—C1	116.4 (2)	C26—C25—H25	119.7
N3—C8—C9	122.7 (2)	O8—C26—C27	125.0 (2)
N3—C8—H8	118.6	O8—C26—C25	114.7 (3)
C9—C8—H8	118.6	C27—C26—C25	120.3 (3)
C10—C9—C14	120.0 (2)	C26—C27—C28	118.9 (2)
C10—C9—C8	122.6 (2)	C26—C27—H27	120.5
C14—C9—C8	117.4 (2)	C28—C27—H27	120.5
C9—C10—C11	119.6 (2)	C29—C28—C27	121.5 (3)
C9—C10—H10	120.2	C29—C28—H28	119.3
C11—C10—H10	120.2	C27—C28—H28	119.3
O4—C11—C12	124.0 (2)	C28—C29—C24	119.2 (3)
O4—C11—C10	115.4 (2)	C28—C29—H29	120.4
C12—C11—C10	120.6 (2)	C24—C29—H29	120.4
C13—C12—C11	119.4 (2)	O8—C30—H30A	109.5
C13—C12—H12	120.3	O8—C30—H30B	109.5
C11—C12—H12	120.3	H30A—C30—H30B	109.5
C14—C13—C12	120.3 (2)	O8—C30—H30C	109.5
C14—C13—H13	119.8	H30A—C30—H30C	109.5
C12—C13—H13	119.8	H30B—C30—H30C	109.5
			
C7—N2—N3—C8	174.3 (2)	C8—C9—C14—C13	178.8 (2)
C22—N5—N6—C23	178.6 (2)	C21—C16—C17—C18	−1.0 (4)
C6—C1—C2—C3	0.0 (4)	C22—C16—C17—C18	178.3 (2)
C7—C1—C2—C3	179.1 (3)	C16—C17—C18—C19	−0.2 (4)
C1—C2—C3—C4	0.6 (4)	C17—C18—C19—C20	1.6 (4)
C2—C3—C4—C5	−0.8 (5)	C17—C18—C19—N4	−177.3 (2)
C2—C3—C4—N1	179.0 (3)	O5—N4—C19—C18	177.6 (2)
O1—N1—C4—C5	2.7 (4)	O6—N4—C19—C18	−1.6 (3)
O2—N1—C4—C5	−177.5 (3)	O5—N4—C19—C20	−1.4 (3)
O1—N1—C4—C3	−177.2 (3)	O6—N4—C19—C20	179.4 (2)
O2—N1—C4—C3	2.7 (4)	C18—C19—C20—C21	−1.7 (4)
C3—C4—C5—C6	0.3 (5)	N4—C19—C20—C21	177.3 (2)
N1—C4—C5—C6	−179.5 (3)	C19—C20—C21—C16	0.3 (4)
C2—C1—C6—C5	−0.5 (4)	C17—C16—C21—C20	1.0 (4)
C7—C1—C6—C5	−179.6 (3)	C22—C16—C21—C20	−178.3 (2)
C4—C5—C6—C1	0.3 (4)	N6—N5—C22—O7	−1.6 (4)
N3—N2—C7—O3	−0.3 (4)	N6—N5—C22—C16	177.5 (2)
N3—N2—C7—C1	179.8 (2)	C21—C16—C22—O7	−179.5 (3)
C6—C1—C7—O3	9.0 (4)	C17—C16—C22—O7	1.2 (4)
C2—C1—C7—O3	−170.1 (3)	C21—C16—C22—N5	1.4 (4)
C6—C1—C7—N2	−171.0 (3)	C17—C16—C22—N5	−177.9 (2)
C2—C1—C7—N2	9.9 (4)	N5—N6—C23—C24	180.0 (2)
N2—N3—C8—C9	179.2 (2)	N6—C23—C24—C25	−178.9 (3)
N3—C8—C9—C10	−6.7 (4)	N6—C23—C24—C29	1.1 (4)
N3—C8—C9—C14	174.2 (3)	C29—C24—C25—C26	−0.2 (4)
C14—C9—C10—C11	1.2 (4)	C23—C24—C25—C26	179.8 (3)
C8—C9—C10—C11	−177.9 (2)	C30—O8—C26—C27	0.1 (5)
C15—O4—C11—C12	3.1 (4)	C30—O8—C26—C25	179.9 (3)
C15—O4—C11—C10	−177.2 (2)	C24—C25—C26—O8	−179.3 (3)
C9—C10—C11—O4	178.9 (2)	C24—C25—C26—C27	0.6 (4)
C9—C10—C11—C12	−1.4 (4)	O8—C26—C27—C28	179.6 (3)
O4—C11—C12—C13	−179.5 (2)	C25—C26—C27—C28	−0.3 (4)
C10—C11—C12—C13	0.9 (4)	C26—C27—C28—C29	−0.4 (4)
C11—C12—C13—C14	−0.1 (4)	C27—C28—C29—C24	0.8 (4)
C12—C13—C14—C9	−0.2 (4)	C25—C24—C29—C28	−0.5 (4)
C10—C9—C14—C13	−0.3 (4)	C23—C24—C29—C28	179.5 (3)

### Hydrogen-bond geometry (Å, °)

**Table:**

*D*—H···*A*	*D*—H	H···*A*	*D*···*A*	*D*—H···*A*
N2—H2N···O9	0.88	1.98	2.839 (3)	166
N5—H5N···O10	0.88	2.08	2.930 (3)	163
C2—H2···O9	0.95	2.23	3.153 (3)	164
C8—H8···O9	0.95	2.45	3.277 (3)	145
C15—H15B···O6	0.98	2.55	3.443 (4)	151
C21—H21···O10	0.95	2.42	3.355 (3)	169
C23—H23···O10	0.95	2.46	3.295 (3)	147
O9—H9A···O7^i^	0.91 (4)	2.26 (3)	3.020 (3)	140 (3)
O9—H9A···N6^i^	0.91 (4)	2.34 (3)	3.093 (3)	140 (3)
O9—H9B···O3^i^	0.92 (3)	1.84 (4)	2.758 (3)	173 (3)
O10—H10A···O7^i^	0.87 (4)	1.99 (4)	2.846 (3)	167 (3)
O10—H10B···O3^ii^	0.80 (4)	2.17 (4)	2.925 (3)	158 (3)
C13—H13···O4^i^	0.95	2.52	3.390 (3)	153
C15—H15A···O6^iii^	0.98	2.59	3.560 (4)	171
C30—H30A···O2^iv^	0.98	2.40	3.322 (4)	156
C30—H30B···O1^v^	0.98	2.59	3.200 (4)	121

Symmetry codes: (i) *x*−1, *y*, *z*; (ii) *x*−1, *y*−1, *z*; (iii) −*x*+2, −*y*+2, −*z*+2; (iv) −*x*, −*y*, −*z*+1; (v) −*x*+1, −*y*, −*z*+1.
